# Natural Deep Eutectic Solvents (NADES): Phytochemical Extraction Performance Enhancer for Pharmaceutical and Nutraceutical Product Development

**DOI:** 10.3390/plants10102091

**Published:** 2021-10-01

**Authors:** Ni Putu Ermi Hikmawanti, Delly Ramadon, Ibrahim Jantan, Abdul Mun’im

**Affiliations:** 1 Graduate Program of Pharmaceutical Sciences, Faculty of Pharmacy, Universitas Indonesia, Cluster of Health Sciences Building, Depok, West Java 16424, Indonesia; ermy0907@uhamka.ac.id; 2 Department of Pharmaceutical Biology, Faculty of Pharmacy and Sciences, Universitas Muhammadiyah Prof. DR. HAMKA, East Jakarta, DKI Jakarta 13460, Indonesia; 3 Department of Pharmaceutics and Pharmaceutical Technology Development, Faculty of Pharmacy, Universitas Indonesia, Depok, West Java 16424, Indonesia; delly.ramadon@farmasi.ui.ac.id; 4 Institute of Systems Biology (INBIOSIS), Universiti Kebangsaan Malaysia, UKM Bangi, Selangor 43600, Malaysia; ibj@ukm.edu.my; 5 Department of Pharmacognosy-Phytochemistry, Faculty of Pharmacy, Universitas Indonesia, Cluster of Health Sciences Building, Depok, West Java 16424, Indonesia

**Keywords:** bioactivity, bioavailability, natural deep eutectic solvents, pharmaceutical formulation, solubility, stability

## Abstract

Natural products from plants were extracted and widely studied for their activities against many disease conditions. The selection of the extracting solvent is crucial to develop selective and effective methods for the extraction and isolation of target compounds in the plant matrices. Pharmacological properties of plant extracts and their bioactive principles are related to their excellent solubility, stability, and bioavailability when administered by different routes. This review aims to critically analyze natural deep eutectic solvents (NADES) as green solvents in their application to improve the extraction performance of plant metabolites in terms of their extractability besides the stability, bioactivity, solubility, and bioavailability. Herein, the opportunities for NADES to be used in pharmaceutical formulations development including plant metabolites-based nutraceuticals are discussed.

## 1. Introduction

Extraction of phytochemical constituents such as phenolics, flavonoids, tannins, alkaloids, terpenoids, steroids, and glycosides from different plant materials (leaves, stems, fruit, seeds, roots, etc.) is an initial step to remove unwanted substances and to isolate phytochemical compounds, which are mostly bioactive natural products used as sources for the development of medicinal ingredients [1]. When carrying out extraction with solvents, the solvent should meet the following criteria: high selectivity (polarity according to the target compound), safety (low toxicity, nonexplosive, and nonflammable), neutral, easy to separate from target compounds, low viscosity (allow ease of transfer mass), low boiling temperature (prevent degradation of compounds), and economical (as cheap as possible). Some of the conventional volatile organic solvents that were used in plant extraction, in order of increasing polarity, include petroleum ether, *n*-hexane, toluene, diethyl ether, chloroform, dichloromethane, ethyl acetate, acetone, *n*-butanol, ethanol, and methanol [2]. Generally, these solvents are used in large quantities, especially when maceration or percolation is used, causing extraction costs to increase. The use of organic solvents is relatively toxic due to improper extraction procedures and possibility of the presence of residual solvents in the extracts produced. The volatility of the solvents makes them potential as air pollutants that contribute to global warming [3]. The use of green solvent could overcome the limitations of conventional organic solvents.

Green solvents are alternative solvents meeting “green” criteria, so they are environmentally friendly and relatively safe [3]. Several advantages of green solvents include that they are easy to prepare (synthesized with environmentally friendly materials and procedures), less hazardous, low energy, recyclable, biodegradable, etc., [4]. Among them, natural deep eutectic solvents (NADES) received great attention from researchers [3]. 

Some natural compounds from plants, such as piperine [5], curcumin [6], or baicalin [7], extracted by the conventional extraction methods showed low solubility, poor stability, low adsorption in the gastrointestinal tract and poor bioavailability [8], which may affect their pharmacological activities. The pharmacological effects of the compounds are often due to their pharmacokinetic behavior, i.e., absorption, bioavailability, distribution, metabolism, and excretion [9]. NADES as an extracting solvent can increase the solubility and bioactivity of these compounds compared to that of conventional solvents [9,10]. The use of NADES can also increase the stability and shelf life of compounds in extracts. In addition, the bioavailability of compounds dissolved in NADES was also reported to increase [11]. 

The present review discusses the applications of NADES to enhance the extraction efficiency of phytoconstituents. The stability, bioactivity, and bioavailability of the plant extracts and their compounds in the solvents and their potential applications in the development of plant-based pharmaceutical formulation and nutraceuticals are also discussed. 

## 2. NADES as an Alternative Solvent

Deep eutectic solvents (DES) are a subclass of ionic liquid (IL). A DES is a eutectic mixture of two or three constituent components, generally interacting through hydrogen bonding, that have a lower melting point than that of each component when combined at the proper molar ratio. The components of these clear liquid mixtures are hydrogen bond acceptors (HBAs) and hydrogen bond donors (HBDs). Choline chloride is the most widely used HBA in the preparation of DES. In addition, betaine, which is an analog of choline chloride, was used. However, making a DES with betaine is considered relatively tricky when compared to that of using choline chloride [12]. Natural deep eutectic solvents (NADES) are a new derivative of DES. NADES are considered "natural" because the constituent components of the eutectic mixture are primary metabolite groups (which are naturally used by the plant itself for survival), such as sugars, organic acids and bases, and amino acids [13]. NADES can be grouped into: (1) ionic liquid NADES (made from an acid and a base), (2) neutral NADES (made from sugars only or sugars and polyalcohol), (3) neutral NADES with acids (made from sugar/polyalcohol and organic acids), (4) neutral NADES with bases (made from sugar/polyalcohol and organic bases), and (5) amino acids-based NADES (made from amino acids and organic acids/sugars) [14]. Many studies report that NADES can extract both hydrophilic and hydrophobic phytonutrient compounds, depending on the constituent components [15]. Interestingly, hydrophilic NADES, such as the aqueous NADES family, can dissolve some lipophilic compounds, unlike conventional solvents such as water [16]. According to Dai et al., 2013, eutectic mixtures with organic acid components generally have the highest polarity, followed by those based on amino acids. On the other hand, a mixture of NADES with polyalcohol and sugar components has a lower polarity than that of the two previous components [13].

Although ILs, DES, and NADES have similar physicochemical properties, currently, NADES are widely employed and meet many criteria as a greener alternative to conventional solvents due to the following properties: (1) their constituent components that are simple in structure and can be found consistently in the market from bulk chemicals; (2) their nonvolatile during the extraction process, which makes NADES usable and recyclable [17]; (3) NADES synthesized with low energy; (4) low toxicity; (5) easily decompose without producing products that are toxic to the environment; (6) NADES have an extraction performance similar to conventional volatile organic solvents; (7) NADES are stable at high temperatures, and (8) are nonflammable [18]. The comparison among ILs, DES, and NADES is provided in Table 1.

Due to the superiority of their “natural” constituent components, NADES support green technology and can be applied in the food, pharmaceutical, and cosmetic industries [18]. These solvents were proven to efficiently produce extracts of plant metabolites with higher yields than those of conventional organic solvents. Because of their green nature, these solvents have increased in demand in a shorter time compared to that of conventional organic solvents. Moreover, the high stabilization and solubilization of NADES can make them excellent candidate to replace conventional organic solvents. However, the main weakness of NADES are the viscosity and variations in the types of constituent components used to synthesize their mixtures [19]. NADES were widely used for extraction from the matrix of natural substances (to obtain a bioactive compound target), as catalysts in enzymatic or chemical processes, and as carriers for insoluble (hydrophobic) compounds for pharmaceutical applications [18]. As extracting solvents, NADES have two mechanisms of action, namely, (1) direct action (interacting with target compounds, usually through hydrogen bonding) and (2) indirect action (damaging the cell wall, releasing the target compound from the plant matrix). In this mechanism, NADES act as a pretreatment solvent [20].

**Table 1:** Comparison among ILs, DES, and NADES [19,21].

Parameters	ILs	DES	NADES
Components	Ionic bonding: organic cation and organic or inorganic anion	Hydrogen bonding: HBA mixed with HBD	Hydrogen bonding: HBA mixed with HBD (primer metabolites that are found in nature)
Melting point	Below 100 °C	Below 100 °C	Below 100 °C
Polarity range	Wide	Wide	Wide
Solubility	High	High	High
Stability	Stable (liquid)	Some can turn back into solids	Some can turn back into solids
Viscosity	High	High	High
Thermolability	Heat resistant	Thermolabile	Thermolabile
Separation	Easy	Hard	Hard
Cost	Relatively high	Relatively low	Relatively low
Preparation of solvent	Hard	Easy	Easy
Toxicity	High	Lower than ILs	Low
Biodegradability	Medium	High	High
Environmentally friendly	Medium	High	High
Recyclability	Yes	Yes	Yes

Cited from: Chemat et al., 2019 and Benvenutti et al., 2019 with modification.

## 3. Solubility of Compounds in NADES

“Solubility is defined as the concentration of dissolved solute in a solvent in equilibrium with undissolved solute at a specified temperature and pressure” [22] is an important aspect of extraction. By promoting or inhibiting specific molecular interactions, solvents can affect a solute’s solubility and stability [16]. In the practical process of plant extraction using solvents, the solubility aspect is crucial to ensure that the process successfully maximizes the yield of plant metabolites. 

The different components of NADES cause differences in polarity, viscosity, and dissolving ability. This is related to the extraction efficiency [23]. According to Cao et al., 2020, high solubility is related to NADES-phytochemical interactions, such as dipole-dipole and hydrogen bonding interactions [11]. Several studies concluded that the extraction of plant metabolites using optimized NADES and extraction process conditions resulted in better yields of bioactive compounds [10] due to the higher solubility of compounds in the NADES (Table 2). One of the important phenolics, namely, chlorogenic acid, was reported to be optimally extracted from several different plants with NADES consisting of various types of HBA-HBD components, including betaine-triethylene glycol (1:2) [24], proline-malic acid (1:1) [25], and choline chloride-1,3-butanediol (1:2) [26] (including other caffeoylquinic acid derivatives (in ratio 1:6) [27]). According to Peng et al., 2016, the increased diffusion and mass transfer are due to an increase in the ratio of polyol components in DES. It was also related to its viscosity and surface tension properties [27]. A study of curcuminoids extraction from *Curcuma longa* L. also reported being optimally extracted with different NADES components, such as citric acid-glucose (1:1) [28], choline chloride-glycerol (1:1) [29], and choline chloride-lactic acid (1:1) [30]. Citric acid is an organic acid with one hydroxyl tricarboxylic acid, which has a greater number of −COOH groups than malic acid (with one hydroxyl dicarboxylic acid) and lactic acid (with one hydroxyl monocarboxylic acid). Thus, citric acid has more opportunities to form hydrogen bonds [28]. The solubility of pure curcumin increased in DES containing choline chloride mixed with an HBA in molar ratio of 1:2, namely, ethylene glycol > glycerol > urea. These DES increased the solubility of curcumin up to 13,000-fold compared to that in water at 25 °C, and 33,000-fold at 40 °C [31]. In the extraction of boldine alkaloids, Torres-Vega et al., 2020 reported that the NADES component with l-proline and oxalic acid (1:1 molar ratio) and 20% water resulted in the best yield compared to that of all other types of NADES used, and it was 8-times more effective than methanol. Alcohol-based NADES, with the same polarity as ethanol, did not produce good yield for the extraction of these compounds compared to that of methanol. The discrepancies between NADES and methanol might be attributed to methanol’s inability to extract partly ionized molecules, where electrostatic interactions could play a key role in their extraction [32].

One of the disadvantages of NADES when compared with that of conventional solvents is their high viscosity [13]. The viscosity of NADES is affected by hydrogen bonding and van der Walls interactions [18]. The high viscosity of NADES can reduce the diffusion coefficients of analytes, leading to low mass transfer and long extraction times. This condition can affect the solubility of the target compound in NADES and have an impact on the effectiveness of the extraction. However, these problems can be solved in two way, i.e., by adding water to the NADES component and by increasing the temperature [35].

In a study conducted by Jurić et al., 2021, among six types of choline chloride-NADES, the lowest viscosity was achieved when using sorbitol as HBD (molar ratio 1:1), followed by fructose as HBD (molar ratio 1:1). However, the choline chloride-sorbitol NADES did not increase extraction efficiency for flavonoid compounds compared to that of the choline chloride-fructose NADES. The authors reported that choline chloride NADES containing a polyalcohol such as glycerol and a sugar as HBD was more effective in extracting flavonoids compared to that of 70% ethanol [36]. Oomen et al., 2020 reported that the addition of 40 and 50% water was able to improve the viscosity of the NADES components, namely, proline-citric acid (1:1, *w/w*) and citric acid-β-alanine (1:1, b/b), respectively. These NADES media was applied to the extraction of *Scutellaria baicallensis* Georgi. *S. baicallensis* contains flavonoid aglycones (baicalein, scutellarein, wogonin, and oroxylin) and flavonoid glycosides (baicalin, wogonoside, and oroxyloside). The solubility of its flavonoid aglycones and flavonoid glycosides in NADES increased by 2–6 times and 1.5–1.8 times, respectively, compared to that of using the aqueous methanol (80%). In this study, it was also reported that too much water (60%) in the NADES matrices (which was hydrophilic) did not effect NADES hydrophilicity, so it did not increase the yield of flavonoid glycosides [34]. Another study conducted by Liu et al., 2019 reported that curcuminoids (bisdemethoxycurcumin, dimethoxy curcumin, and curcumin), yellow pigment compounds from *Curcuma longa* L., were highly soluble in NADES with citric acid-glucose (1:1) added to 15% water compared to that of conventional solvents, namely, ethanol and methanol. The addition of too much water (>15–35%) can weaken hydrogen interactions between the solvent and the target compound, resulting in reduced yields of curcuminoids. The optimal NADES for the extraction of natural compounds has a viscosity that is neither too high nor too low [28]. This shows that the capacity of NADES to dissolve target compounds in plants can be modified by adjusting the type of NADES components at the appropriately mixed ratio molar and water content in NADES [11].

Besides selection of suitable NADES to increase the solubility of the target compound in the extraction process, it is also necessary to consider the extraction conditions, including the extraction method. Several methods based on heating, ultrasonic energy, and microwave radiation were proposed to enhance the mass-transfer of target molecules from the solid sample phase to the DES phase and minimize equilibrium time [35]. Wang et al., 2017 conducted a study to choose an appropriate DES for the effective extraction of chlorogenic acid from blueberry leaves. Eight different types of DES were produced and tested. Choline chloride-1,3-butanediol (2:1) NADES was chosen and utilized in the process of optimizing extraction conditions. The tested solvent optimization parameters included the water content, liquid-solid ratio, temperature and time. The extraction of blueberry leaves with NADES assisted by negative pressure cavitation extraction (NPCE) provided good extraction efficiency of chlorogenic acid compared to other commonly used extraction methods (NPCE > MAE > HRE > UAE). Under this condition, the extraction was carried out at a low temperature (59 °C) in only 24 min [26]. Another study by Peng et al., 2016 found that using the MAE method to extract five types of phenolics (chlorogenic acid, caffeic acid, 3,4-dicaffeoylquinic acid, 3,5-dicaffeoylquinic acid, and 4,5-dicaffeoylquinic acid) from *Lonicera japonica* Thunb. flos in NADES was more efficient than other methods (MAE > UAE and HRE). The MAE extraction requires a temperature of 60 °C and was carried out within 20 min [27]. Compared to that of MAE, extraction with UAE takes longer [37]. Although there are many reports on the magnitude of the recovery of phenolic target compounds with MAE [38], it is also highly dependent on the physicochemical properties of the NADES used and the chemical molecules of the target compounds. Thus, choosing the appropriate combination of the NADES and extraction method will undoubtedly be able to increase the solubility of the target compound in NADES. This extraction efficiency is related to the reducing in the volume of solvent used for extraction, operating costs, and extraction time [38].

## 4. Stability of Compounds in NADES

The types of NADES components (with different composition) produce various physical properties that may lead to different extraction capabilities [39]. Among 100 NADES tested, most of them showed stability at high temperatures (>200 °C) [13]. The properties of NADES, such as chemical and thermal stability, are also associated with the storage of natural compounds [17]. The stabilization ability of NADES for target compounds is essential for further applications in food, cosmetics, and pharmaceutical formulations [40]. One of the uses of NADES is for stabilization of plant natural pigments [41]. A summary of the applications of NADES for increasing the stability of plant metabolites is shown in Table 3. For example, the stability of carthamin, a prominent natural red pigment component derived from safflower (*Carthamus tinctorius* L.), was investigated by Dai et al., 2014. In an aqueous solution, the compound is highly unstable; therefore, it is generally extracted using an alkaline solution. The authors reported that in xylitol-choline chloride NADES at 40 °C, the half-life (t1/2) of carthamin was 5-times more stable compared to 60 °C, but it was 8-times more stable in water at the same temperature. Thus, xylitol-choline chloride exerts a protective effect on carthamin against heating compared to water. The stability of carthamin to light in a sugar-based NADES solution was also studied at room temperature. Over a 15-day period, the solution was exposed to light for 24 h compared to the dark. The results suggest that glucose-choline chloride and sucrose-choline chloride NADES were better solvents than 40% ethanol or water for carthamin stabilization. These NADES also help to keep carthamin stable during storage. Carthamin could be kept in choline chloride NADES containing sucrose for at least 30 days at 4 °C and at least 90 days at −20 °C. The high viscosity of NADES containing sugar and the molecular interactions between the solvent and the carthamin were most likely responsible for the protective effects against degradation caused by heat, light, and time, therefore enhancing bio-component stability [40].

Jeliński et al., 2019 tested the stability of curcumin powder, a yellow pigment derived from turmeric, in methanol and choline chloride-glycerol NADES following exposure to artificial sunlight. After 120 min, the concentration of curcumin in methanol solution fell to 5% of its starting level, whereas in NADES solution, curcumin was stable, and there was no degradation [29]. Similarity, according to Liu et al., 2019, curcumin is more stable in NADES (citric acid-glucose) than ethanol and methanol at 80 °C. To promote the good stability of curcumin in NADES, storage should be at 4 °C instead of 25 °C. The high stabilization ability of NADES might be caused by the interaction between curcuminoids and NADES molecules. The oxygen atom of the hydroxyl group formed hydrogen bonds with the two components of citric acid-glucose, which could protect curcuminoids from oxygen, which could cause oxidative damage [28]. Similar opinion was expressed by Dai et al., 2016. Using cyanidine as a marker, the authors investigated the influence of the solvent, temperature, and storage duration on anthocyanin stability in lactic acid-glucose NADES. The authors found that at 60 °C, cyanidin was more stable in NADES than in acidified ethanol, and it was stable in both solvents for 1.5 h in the dark at 40 °C. Meanwhile, at 60 °C, the half-life time (t1/2) of cyanidin in NADES was more than threefold longer than that of acidified ethanol. As a result, the solutions should be kept at 4 °C in the dark for one week after preparation and then evaluated. The solutions could be kept at −20 °C in the dark for up to 3 months for long-term storage. The intermolecular interaction of the NADES components with cyanidin (especially hydrogen bonding with hydroxyl and carboxyl groups) could decrease solute molecule mobility and oxygen contact at the NADES-air interface. It could reduce the possibility of oxidative damage [39]. The stability of other hydrophobic pigments such as β-carotene in NADES was studied by Stupar et al., 2021. The authors reported that fatty acid NADES containing caprylic acid-capric acid (3:1) optimally extracted β-carotene from pumpkin (96.74 μg/mL). These NADES increased the solubility of β-carotene up to 200.77 μg/mL, compared to 107 μg/mL with *n*-hexane. In addition, the NADES extract also showed high stability during storage for 180 days in the dark. β-carotene from pumpkin in NADES has good stability if stored at 4 °C. Hydrogen bonding interactions between solute and solvent molecules and pH are related to the stability of carotene in solution [42].

The effect of storage duration on phenolic degradability in DES extract compared to that of an ethanolic extract of rosemary at 25 °C was monitored by DPPH activity testing at 72 h intervals. Using the Weibull model, the half-life (t1/2) of DES extracts ranged from 7 to 49 days. The kinetic constant (k_α_) and the t1/2 of ethanol extract were significantly lower than those of DES extracts (with choline chloride-glycerol) [43]. In another study, at 80 °C for varying times, four phytochemicals, namely, andrographolide, baicalin, curcumin, and oleanolic acid, showed better stability in NADES solutions than 0.9% sodium chloride solution, which was used as a control due to its common use as a solvent in drug delivery [11]. Epigallocatechin-3-gallate (EGCG) may require changing manufacturing and storage processes that are affected by temperature and time. Jeong et al., 2017 evaluated the stability of EGCG in NADES during storage. The authors reported that the decrease in EGCG levels in NADES was smaller than in the conventional solvent used for comparison [44]. This means that interactions between NADES and phytochemicals can prolong phytochemical shelf life. The author of this study underlined that the stabilizing capacity of NADES could be enhanced by lowering its water content (increased viscosity). This is affected by the strong hydrogen bonding between the solutes and NADES [11].

**Table 3:** Application of DES/NADES in improving stability of plant metabolites.

Plants	Bioactive Compounds	Selected DES/NADES (Molar Ratio in mol/mol) and Optimal Condition	Conventional Solvents for Comparison	Results	Refs.
Safflower (*Carthamus tinctorius* L.)	Carthamin	NADES that contain sugar, such as glucose-choline chloride, sucrose-choline chloride, xylitol-choline chloride; heating and stirring at 40 °C for 30 min.	Water and 40% ethanol	Natural safflower pigment was more stable in NADES that contained sugar than in water or 40% ethanol solution under various conditions (high temperature, light, storage time).	[40]
Flower petals of *Catharanthus roseus* (L.) G.Don	Cyanidin (anthocyanins)	Lactic acid-glucose (5:1) using UAE for 30 min.	3% formic acid in methanol	The extraction capacity of the selected NADES for anthocyanins was equal to that of the conventional solvent, but it had at least three times the stabilizing capacity for cyanidins.	[39]
Roselle (*Hibiscus sabdariffa* L.)	Anthocyanins	Sodium acetate-formic acid (molarity ratio of 1:2); UAE at room temperature for 20 min.	Distilled water, 70% ethanol, and 80% methanol	Anthocyanins were more stable in the selected NADES with 0% additional water.	[45]
*Curcuma longa* L.	Curcuminoids	Citric acid-glucose (1:1) and 15% water content; matrix/solvent ratio was 0.1/10 g/mL; 50 °C and 30-min extraction time with constant stirring.	Ethanol and methanol	At 80 °C, curcuminoids were more stable in NADES than in ethanol and methanol. The stability of curcuminoids test suggest that 4 °C, rather than 25 °C, is the best temperature for storing curcuminoids in NADES.	[28]
		Choline chloride-glycerol (1:1), mechanical stirrer for 24 h.	Methanol	Curcumin is more stable in NADES solution than in methanol.	[29]
Rosemary (*Rosmarinus officinalis* L.)	Phenolics	Choline chloride-lactic acid (3:1) and 10% water; at 40 °C, 50–60 Hz, for 120 min using UAE.	Pure ethanol	Ethanolic extracts had a lower kinetic constant (k_α_) and half-life (t1/2) than NADES extracts, indicating that the NADES has a higher stabilizing capability than ethanol.	[43]
Mulberry	Anthocyanins (Cyanidin-3-glucoside)	Choline chloride-lactic acid (1:2) with 20% water; using UAE for 10 min at 40 °C in the absence of light.	80% ethanol and 0.4% HCl, lactic acid	Anthocyanin stability enhancement occurred in NADES solution compared to the tested acidified ethanol at 80 °C. If stored at −20 °C, only about 7% anthocyanins were lost in NADES, while in acidified ethanol, up to 20% were lost. After 3 months of storage at 4 °C and 25 °C, the anthocyanin concentration in NADES was 1.2- and 1.7-fold greater than that in acidified ethanol.	[46]
Green tea (*Camellia sinensis* (L.) Kuntze)	Catechins (epigallocatechin-3-gallate (EGCG))	Betaine-glycerol-glucose (4:20:1) and 30% water; using UAE for 45 min at ambient temperature.	Water, methanol, 70% methanol, and 70% ethanol	NADES is more efficient in EGCG extraction than 70% ethanol. The decrease in EGCG levels in NADES was smaller than in the comparison conventional solvent.	[44]

## 5. Bioactivity of Compounds from NADES Extract

The solubility and stability of plant bioactive compounds are among the essential criteria for ensuring an adequate degree of bioactivity for beneficial health effects [47]. Many studies identify these parameters as key to the success of the bioactivity test of the NADES extract from plants. However, the results are not always correlated and are not easy to interpret, so further investigation is needed. Most of the bioactivity tests carried out on NADES extracts are in vitro tests, such as antioxidant activity, enzyme inhibition, and cell culture tests. Most of these assays of NADES extracts yielded satisfactory results compared to that of conventional solvent extracts [48]. Table 4 summarizes several studies on the bioactivity assay of NADES extracts discussed in this review.

Antioxidant molecules, such as polyphenols, flavonoids, and carotenoids, can be effectively extracted using NADES. Evaluation of the antioxidant activity of NADES extract can be conducted in the form of expensive kits or cell culture [10]. In a study by Mansinhos (2021), the antioxidant activity against 1,1-diphenyl-2-picrylhydrazyl (DPPH●) and 2,2′-azinobis-(3-ethylbenzothiazoline-6-sulphonate) (ABTS^●^^+^) radicals of NADES (choline chloride-urea) extract showed better results than extracts in conventional solvents (water, 80% ethanol and methanol). However, among the NADES variations tested in the study, this NADES extract (total phenolics = 11,968 g/g of extract) did not contain the highest total phenolic content. The highest total phenolic content was found in the proline-lactic acid extract (total phenolics = 12,709 g/g of extract). This extract showed good activity in oxygen radical absorbance capacity (ORAC) and ferric reducing antioxidant power (FRAP) assays. This might be connected to the quantity of individual phenolic components in the NADES extract. The extract in choline chloride-urea contained the most fertaric acid compared to that in other NADES and conventional solvents. In addition, the extract in choline chloride-urea was also effective in extracting ferulic acid and rosmarinic acid from *Lavandula pedunculata* subsp. *lulsitanica* (Chaytor) Franco. [49]. The antioxidant extracts from *Mangifera pajang* Kosterm fruit wastes were much more soluble in choline chloride-ascorbic acid (2:1) NADES with 10% water content than in water. There might be interactions in the form of hydrogen bonding between the hydroxyl groups of ascorbic acid and choline chloride. This interaction between NADES components helps to increase the solvation capacity, observed in the ^1^H Nuclear Magnetic Resonance (NMR) analysis. As a liquid phase, NADES is essential for dissolving hydrophilic bioactive chemicals. The extract produced from NADES also increased its antioxidant capacity compared to the use of water as the extraction solvent [47]. Furthermore, when compared to that of antioxidant extracts in water, the application of choline chloride-ascorbic acid demonstrated increased capacity to protect antioxidant components against high temperature and pH, with the half-life prolonged by 4.17–25% [50]. However, Gullón et al., 2019 reported that extraction using DES with choline chloride and ethylene glycol (1:2) from *Eucalyptus globulus* Labill. did not increase phenolic antioxidant activity against DPPH radicals compared to that of extraction with aqueous ethanolic. The author stated that a thorough evaluation is needed using other types of NADES, which can certainly be used to increase the efficiency of DES [51]. The antioxidant power of the NADES extract is related to intermolecular interactions, especially hydrogen bonds that stabilize the target compound. According to Jurić et al., 2021, it is important to evaluate the pure NADES system tested to determine whether NADES donates a hydrogen atom to the DPPH radical, which is indicated by the occurrence of discoloration in the DPPH solution [33]. For example, NADES with sugar content can affect the antioxidant test results against DPPH. Their study also proved that the pure NADES system tested did not have the ability to reduce ferric ions. Thus, it was concluded that the bioactive compound of the peppermint extract in the NADES that they tested provided an indication of the antioxidant activity against DPPH radicals and the ability of reduce ferric ions [36]. Additionally, according to Doldolova et al., 2021, the antioxidant capacity of the extract obtained with NADES can be suitably tested using the cupric ion (Cu^2+^) reducing antioxidant power assay (CUPRAC) method, which selectively oxidizes antioxidant compounds only. Thus, using sugar components, citric acid, or amino acids that are susceptible to interference during the analysis stage can be resolved [33]. This certainly proves that to obtain an optimal increase in antioxidant activity, it is necessary to optimize the selection of the type of eutectic mixture to dissolve the target compound and, of course, optimize of extraction.

The biological effect of NADES extract on the inhibition of certain enzymes was also studied by many researchers. In testing the inhibition of enzymes (acetylcholinesterase, butyrylcholinesterase, and tyrosinase) involved in neurodegenerative diseases, it was shown that except for proline-lactic acid against butyrylcholinesterase, all organic-acid-based NADES extracts (with strong acid pH) showed the greatest potential to inhibit acetylcholinesterase and butyrylcholinesterase. However, the extract of *L. pedunculata* in this study did not provide potent inhibitory activity against tyrosinase [49]. In comparison, according to Oktaviyanti et al., 2019, at a concentration of 50 mg/mL, bioactive compounds extracted from *Ixora javanica* (Blume) DC flower with choline chloride DES containing organic acids as HBDs (such as lactic acid (at molar ratio 1:2) and malic acid (at molar ratio 1:1)) inhibited the tyrosinase enzyme by 65% and 72%, respectively [52]. Another enzyme inhibitory activity test, namely, dipeptidyl peptidase IV (DPP IV), was carried out on NADES extract by Ahmad et al., 2020. The authors reported that cinnamon and sappan wood were extracted, each with choline chloride-glycerol in different molar ratios. The results showed that the IC_50_ value of the NADES extract from each sample was not better than that of the ethanolic reflux extract (82.0 μg/mL). However, the NADES extract from a combination of the two plant samples (36.5 μg/mL) had better IC_50_ value than the ethanolic extract [53]. According to Sakti et al., 2019, choline chloride-glycerol is a hydrophilic NADES, and its polarity is greater than that of water. The difference in the molar ratio of the HBA and HBD components of NADES greatly contributes to the extraction capacity of the target compounds (brazilin in sappan wood and trans-cinnamaldehyde and coumarin from cinnamon) [54].

Grozdanova et al., 2020 reported that the extraction of aerial parts of *Sideritis scardica* Griseb. and *Plantago major* L. using choline chloride-glycerol (1:2) with 30% water and citric acid-1,2-propanediol (1:4) showed high antibacterial activity and minimal genotoxicity and cytotoxicity. According to the authors, the use of organic acids, such as citric acid, in the NADES media was one of the factors that contributes to the increased antimicrobial activity. NADES that contain citric acid have a polarity close to that of 70% ethanol. The author reported that the obtained phenolic content was better extracted with choline chloride-glucose (5:2) with 30% water than other tested types of NADES and 70% ethanol. However, it results show that this NADES extract was inactive against all tested microorganisms [55]. A similar finding was reported by Jurić et al., 2021. The authors testing the ability of peppermint extracts in NADES to inhibit bacterial growth and investigated the toxicity of NADES only (without extract). According to the result, peppermint extract in NADES containing organic acid components, such as malic acid and citric acid, showed higher antimicrobial activity than other extracts against Gram-negative bacteria (namely, *Pseudomonas aeruginosa*, *Escherichia coli* and *Salmonella enterica*) and Gram-positive bacteria (*Staphylococcus aureus*). Likewise, pure NADES containing both types of acids showed high antimicrobial activity against the test bacteria. This is presumably due to the acidic nature of NADES (low pH), which affects the optimal pH for bacterial growth. Conversely, pure NADES containing sugar and alcohol as HBD did not produce an antimicrobial effect against *E. coli* and *S. aureus*. It is suggested that the selection of HBD species can be the key to determining the toxicity of NADES to the test bacteria [36].

**Table 4:** Application of DES/NADES to improve bioactivity of plant metabolites.

Plants	Bioactive Compounds	Extraction Methods Using DES/NADES	Conventional Solvents for Comparison	Results	Refs.
Rosemary (*Rosmarinus officinalis* L.)	Phenolics	Choline chloride-lactic acid (3:1) and 10% water at 40 °C, 50–60 Hz, for 120 min using UAE.	Pure ethanol	DES-based extracts presented higher antioxidant capacity than the alcohol extract (DPPH and FRAP assays).	[43]
*Curcuma longa* L.	Curcuminoids	Citric acid-glucose (1:1) and 15% water; matrix-solvent ratio was 0.1/10 g/mL, at 50 °C for 30 min with constant stirring.	Ethanol and methanol	Radical scavenging activity (RSA) of curcuminoids was greater in NADES (87.0%) than in methanol (86.3%) but lower than ethanol (90.0%).	[28]
Fruit Wastes (peel and kernel) of *Mangifera pajang* Kosterm.	-	Choline chloride-ascorbic acid and 10% water.	Water	Antioxidant capacity against DPPH free radicals of the extract in NADES increased (1.3–14.64%) compared to the antioxidant extract in water.	[47]
*Ixora javanica* (Blume) DC. flower	Flavonoids and anthocyanins	Choline chloride-propylene glycol (1:1) for 5 min at 57 °C; matrix-solvent ratio was 0.02 g/mL, using UAE.	Ethanol	The selected NADES extract had higher inhibition of DPPH free radical and tyrosinase activity than ethanolic extract.	[52]
Elderberry (*Sambucus nigra* L.) Flowers	Polyphenols	l-lactic acid-glycine and 15% water; solvent-matrix ratio was 60 mL/g, and stirring at 200 rounds per min.	Deionized water, 60% ethanol, and 60% methanol	The NADES extract showed the highest reducing power and antiradical activity toward DPPH than conventional solvent extracts.	[56]
Onion (*Allium cepa* L.) peel	Phenolics	Choline chloride-urea-water (1:2:4); MAE at 100 Watt, solvent-matrix ratio was 54.97 mL/g, for 15.03 min.	70% aqueous methanol with Soxhlet extraction	Under the optimized MAE conditions, the NADES extract had the higher antioxidant activity in FRAP assay (636.18 μmol AAE/g dry weight) than extract from conventional solvent.	[48]
*Lavandula pedunculata* subsp. *lulsitanica* (Chaytor) Franco	Phenolic	Choline chloride-urea (1:2); UAE for 60 min.	Water, 80% ethanol and methanol	The extract in selected NADES showed higher antioxidant activity (DPPH and ABTS assay) than extract in the conventional solvent. In addition, this extract also provides good activity against acetylcholinesterase (AChE) and butyrylcholinesterase (BChE) enzymes, but it was less potent against tyrosinase (Tyr).	[49]
Cinnamon bark (*Cinnamomum burmannii* (Nees & T.Ness) Blume))	trans-cinnamaldehyde, coumarin, and trans-cinnamic acid	Choline chloride-glycerol (2:1) with 20% water content; matrix-solvent ratio was 1:4 *w/w*; UAE, 35 W, 42,000 Hz for 30 min.	96% ethanol	NADES extraction from cinnamon had DPP IV inhibitory activity of 205.0 μg/mL.	[53]
Sappan wood (*Caesalpinia sappan* L.)	Brazilin	Choline chloride-glycerol (1:2) 47.6 % water content; matrix-solvent ratio was 2:1 *w/w*; UAE, 35 W, 42,000 Hz for 50 min.	96% ethanol	NADES extract from sappan wood had DPP IV inhibitory activity 1254.0 μg/mL.	[53]
The aerial part of *Sideritis scardica* Griseb. *and Plantago major* L.	Phenolics and flavonoids	Choline chloride-glycerol (1:2) with 30% water; citric acid-1,2-propanediol (1:4); extraction using UAE without heating for 1 h.	70% ethanol	NADES extracts were effective against *Streptococcus pyogenes, Escherichia coli, Staphylococcus aureus,* and *Candida albicans*. They also have minimal genotoxicity and cytotoxicity.	[55]
Grape pomace from *Vitis vinifera* L. and Olive pomace	Phenolics	Choline chloride-citric acid (2:1) with 30% water; extraction using UAE and MAE.	70% ethanol	In HeLa and MCF-7 cells, the NADES extracts of both pomaces were more cytotoxic than the ethanolic extract.	[57]
Astragali Radix (Mikvetch Root)	Flavonoids and saponins (acetylastragaloside I)	Glucose-fructose-sucrose (32:32:5 by weight) and water.	-	The NADES-imitated honey enhanced the quantities of active components as well as their immunological effectiveness.	[58]
Grape skin (*Vitis vinifera* L.)	Phenolics	Choline chloride-malic acid (1:1) with 30% water; UAE, at 65 °C, for 50 min.	70% methanol	The NADES extract had the highest antioxidant (ORAC values were 371 μmol TE/g dry weight) and antiproliferative activities (about 20% of the cells were viable.).	[59]
*Mentha piperita* L.	Phenolics	Choline chloride-malic acid (1:1) Choline chloride-sorbitol (1:1) Choline chloride-fructose (1:1)	70% ethanol	The NADES containing organic acids showed bacterial growth inhibition at a lower concentration than conventional solvent. In FRAP test, most NADES extracts were able to neutralize DPPH radicals better than 70% ethanol and had a similar capacity to decrease Fe^3+^ to Fe^2+^ ions.	[36]

Several NADES (without active compounds from plants) were investigated for their low cytotoxicity in HeLa and MCF-7 cells (EC_50_ > 2000 mg/L) including choline chloride-glucose (2:1), choline chloride-fructose (1.9:1), choline chloride-xylose (2:1), choline chloride-glycerol (1:2), and choline chloride-malic acid (1:1). Five extracts of grape skin obtained using these NADES showed higher cytotoxicity against cancer cells than the corresponding methanol extracts, with choline chloride-malic acid having the greatest inhibitory activity [59]. In another study, Panić et al., 2019 tested the antiproliferative activity of NADES extracts of grape pomace and olive pomace in two human tumor cell lines. Choline chloride-citric acid (2:1) was used in NADES containing 30% water. The authors reported that in HeLa and MCF-7 cells, NADES extracts from both pomaces were more cytotoxic than ethanolic extracts. This result is related to the high phenolic content of the NADES extract and the low pH value of choline chloride-citric acid used as the NADES component in the extraction of both pomaces [57]. In HepG2 (human liver cells) and MCF-7 (human breast cancer cells) cell lines, Cao et al., 2020 investigated the anti-proliferative activities of four plant chemical compounds, namely, baicalin (flavonoid), curcumin (polyphenolic), andrographolide (diterpene lactone), and oleanolic acid (pentacyclic triterpenoid), which were dissolved in different NADES. Choline chloride-levulinic acid (1:2) was identified as the best NADES for dissolving baicalin and curcumin, while acetamide-1-propanol (1:8) was best for oleanolic acid dissolution, and lactic acid-1-propanol (2:1) was best for andrographolide dissolution. The chemical (such as polarity) and physical (such as viscosity) properties were considered in the selection of NADES. According to the authors, the use of liquid components was more beneficial because they produced a mixture with a low viscosity. The results showed that the antiproliferative activity of phytochemical compounds dissolved in NADES were 1.22–4.19 times stronger compared to when they were when dissolved in DMSO, which was used as a control. The authors also reported increased solubility and stability of plant metabolites in NADES. This improvement is closely related to the hydrogen bonding and dipole-dipole interactions between NADES and these phytochemicals [11].

In plant extraction, organic-acid-based NADES often produces good yields of hydrophilic compounds, including phenolic groups [53,55]. This is of interest because it is related to an increase in the biological activity of the plant extracts tested. However, the high acidity of organic acids needs special attention because it can sometimes be a problem for in vitro activity testing, both in enzymatic assays and in tests on cell culture. It is important to underline that the assessment of biological effects requires a control as a comparison so that it can be determined whether the effect is due to the active compound, the pure NADES, or the interaction of the two. It is no less important to test the toxicity of the NADES extracts. If proven toxic, then the biological effect of the bioactive compound could be impaired [10].

## 6. Bioavailability of Compounds in NADES

As previously mentioned, some plant metabolites have low water solubility. This poses a risk of becoming an obstacle in developing active plant metabolites as drugs due to their low bioavailability. Drug bioavailability is the primary factor in the success of a delivery system [60]. Low bioavailability can result in an excessively high dose and the possibility of adverse side effects [11]. NADES have the potential to be used as administration vehicles for pharmaceutical products [61]. Several studies investigated the effect of using NADES as a solvent and a conducting agent for certain compounds. Increased solubility can increase the oral bioavailability of the compound [9]. A summary of the studies of the bioavailability of bioactive compounds in NADES is presented in Table 5.

The bioavailability of curcumin in NADES (choline chloride-glycerol) was tested by measuring its solubility in gastrointestinal fluids, namely, Fasted State Simulated Intestinal Fluid (FASSIF) and Fasted State Simulated Gastric Fluid (FASSGF), by Jelinski et al., 2019. The results reveal that the solubility of curcumin in NADES was approximately 3.5 times more for FASSGF and approximately 2 times greater for FASSIF than curcumin powder in FASSGF and FASSIF, used as references. These results show that NADES could be used as an agent to enhance curcumin’s solubility for oral administration to individuals [29]. In an in vivo pharmacokinetic study of berberine alkaloids in NADES, the results showed that blood levels of berberine increased from 2- to 20-times in rats 6 h after they were given NADES solution containing berberine at a dose of 50 mg/kg by gavage compared to a berberine hydrochloride water suspension. The best solubility of berberine in NADES at 22 °C was obtained in a proline-urea mixture (2:1) (12.3 mg/mL) and proline-malic acid-lactic acid-water (1:0.2:0.3:0.5) (25.0 mg/mL). Thus, there is a relationship between the observed increase in bioavailability and the solubility properties of various eutectic mixtures [61]. Another pharmacokinetic study was also carried out by Faggian et al., 2016 using rutin dissolved in NADES. The results showed that the levels of rutin in blood plasma increased over a longer period in Balb/c mice given rutin in NADES containing proline-glutamic acid (2:1) compared to an oral dose of a water suspension [62].

Funari et al., 2019 reported that honey and NADES made from choline chloride-propylene glycol or lactic acid could replace ethanol and water for the extraction of green propolis [65]. Dai et al., 2020 evaluated the quality-enhancing effects of honey on Astragali Radix compared with NADES. They results show that the physicochemical characteristics of honey and NADES were similar. Both could increase the dissolution concentration of active compounds, such as total flavonoids and saponins. The plasma concentration of calycosin-7-O-β-D-glucoside (isoflavonoid) in rats was increased 12 h after oral administration of this compound, which was previously dissolved in honey and NADES. Tong et al., 2021 found that l-proline-acetamide NADES had a higher extraction efficiency on hydroxysafflor yellow A (HSYA) (32.43 mg/g) and anhydrosafflor yellow B (ASYB) (8.44 mg/g), the main bioactive compounds from the dried flower of *Carthamus tinctorius* L., which was 21.3% and 46.6% higher than that of water. Plasma levels of the compounds in male Sprague–Dawley rats was increased after oral administration at a dose of 2 g extract/kg compared to an aqueous extract suspension. This indicates that HSYA and ASYB were promptly absorbed and then eliminated in 12 h [64].

## 7. Applications of NADES in Pharmaceuticals and Nutraceuticals 

The ability of NADES to increase the solubility, stability, bioactivity, and bioavailability of target compounds was discussed previously. NADES is capable of extracting wide ranges of bioactive target compounds, both hydrophilic and hydrophobic. Another advantage of NADES is that they are safe (low toxicity, nonvolatile, and nonexplosive). These properties makes NADES potentially useful in the food industry, pharmaceutical and nutraceutical formulations, and cosmetics [14]. A transdermal drug delivery system is an intriguing and promising medicinal use of DES/NADES [41].

Cynaropicrin is a bitter compound from the leaf extract of *Cynara cardunculus* L. which has the potential as a phytochemical-based nutraceutical source. This compound was successfully obtained with high yields using NADES which contains decanoic acid- tetrabutylammonium chloride and 70 % water, at 25 °C, for 60 min, and solid-liquid ratio of 1:30 [66]. Application of NADES in the pharmaceutical formulation was carried out by Liu et al., 2018. The authors reported that the NADES could assist the formulation of curcumin (a hydrophilic molecule) in the form of a hydrogel, which was then used as a model to develop crude extract of *Schisandra chinensis* (Turcz.) Baill. The selected NADES components were mannose-dimethyl urea-water (2:5:5). This NADES had superior solubilizing power for curcumin. The process of making the hydrogel was carried out by loading curcumin into chitosan-alginate beads [67]. Additionally, Basar et al., 2020 reported that through the use of emulsion electrospraying and 10% DES containing choline chloride-butadienol, β-carotene was effectively encapsulated in whey protein concentrate capsules. When compared to that of free β-carotene (in the *n*-hexane solution), the capsules had the highest carotenoid loading capacity and outstanding photo-oxidation stability after 180 min of light exposure. The application of DES as the solvent enhance the solubility, stability and bioavailability of the compound in encapsulation formulation [68]. The results obtained provide fundamental value in the bioactive encapsulation.

Their nonvolatile nature and difficulty removing directly after extraction make NADES an alternative solvent that can be applied, with the potential to become a natural part of pharmaceutical [3] and nutraceutical formulations [24,62]. Thus, it can reduce the unit operations of the long series of processes involved in making medicines from natural ingredients. For the future, NADES applications in food, pharmaceutical, and nutraceutical formulations still need to be researched and developed.

## 8. Conclusions

In summary, several studies reviewed in this article showed that NADES have great potential as an alternative solvent and is especially successful in increasing the extraction ability, solubility, stability, bioactivity, and bioavailability of bioactive compounds in plants. NADES selected as an alternative solvent must have high selectivity towards biological targets with the lowest possible toxicity. It is necessary to consider the chemical and physical aspects of NADES components when carrying out the synthesis of the eutectic mixture to optimize the solubility and the stability of the bioactive compounds. Selection of the suitable type of NADES and optimizing the extraction process can lead to improved plant metabolite extraction performance while meeting the principle of green extraction compared to that of conventional solvents. These factors will impact future scale-up processes for NADES applications in the food, pharmaceutical, and nutraceutical industries. The abilities of NADES are very promising to help solve important problems development of pharmaceutical and nutraceutical formulations. Experiments confirming NADES as a safe and environmentally friendly solvent also needs to be followed by an in-depth and thorough study of the toxicity and recovery process of the compounds in the NADES extract. However, there is still much to be studied and scientifically proven for these solvents before they are actually used in the food, pharmaceutical, and nutraceutical sectors.

## Figures and Tables

**Table 2:** Application of DES/NADES in improving solubility of some plant metabolites.

Plants	Bioactive Compounds	Selected DES/NADES (Molar Ratio in mol/mol); Optimal Conditions	Conventional Solvents for Comparison	Results	Refs.
Spent Coffee ground	Chlorogenic acid	Betaine-triethyleneglycol (1:2) and 30% water and matrix-solvent ratio was 1:15 (g/mL) using ultrasonication at 65 °C for 20 min.	70% methanol	Compared to choline chloride-based NADES and 70% methanol, betaine-triethylene glycol (1:2) was demonstrated to be the most powerful in extracting total chlorogenic acids from spent coffee grounds.	[24]
Herba artemisiae scopariae (*Artemisia argyi* H.Lév. & Vaniot)	Chlorogenic acid	Proline-malic acid (1:1) using pre-treatment with ultrasonic for 30 min followed by continuous stirring for 2 h.	Water and ethanol	In comparison to other NADES and conventional solvents, the chosen NADES with the highest solvation free energy had the best impact for chlorogenic acid extraction.	[25]
Blueberry leaves	Chlorogenic acid	Choline chloride-1,3-butanediol (1:2) using negative pressure of −0.07 Pa; solvent-matrix ratio was 17.01 mL/g, at 59.03 °C, for 24.12 min.	-	NPCE was used for extraction The method is efficient for extraction of Chlorogenic acid (NPCE > MAE > HRE > UAE).	[26]
*Lonicera Japonica* Thunb.	Chlorogenic acid, caffeic acid, 3,4-dicaffeoylquinic acid (3,4-DCQA), 3,5-dicaffeoylquinic acid (3,5-DCQA), and 4,5-dicaffeoylquinic acid (4,5-DCQA)	Choline chloride-1,3-butanediol (1:6) with 10% water; solvent-matrix ratio was 9 mL/g, at 60 °C for 20 min using MAE.	-	MAE was an efficient method of extracting five phenolic compounds from *L. japonica* flos (MAE>UAE and HRE).	[27]
*Curcuma longa* L.	Curcuminoids	Citric acid-glucose (1:1) and 15% water; at 50 °C, solvent-matrix ratio was 0.1/10 g/mL, for 30 min with constant stirring.	Ethanol and methanol	Citric-acid-glucose yielded the most curcuminoids, followed by malic acid-glucose (1:1) > ethanol > methanol. Curcumin at 21.18 mg/g, bisdemethoxy curcumin at 16.54 mg/g, and demethoxy curcumin at 15.12 mg/g.	[28]
		Choline chloride-glycerol (1:1), mechanical stirrer for 24 h.	Methanol and water	The percentage of curcuminoids extracted from three commercial turmeric powders from different suppliers in NADES (0.94–1.26%) was higher than in water (0.06–0.08%). However, it is still lower than methanol (5.05–5.09%).	[29]
		Choline chloride-lactic acid (1:1) and 20% water content; 5% solid loading; at 30 ± 2 °C in 20 min using UAE.	-	The maximum curcuminoids yield was 77.13 mg/g; The solubility of curcuminoids was 13.7 mg/mL.	[30]
		Sucrose-lactic acid-water (1:5:7) 15–20 min, solvent-matrix ratio was 14.5–16.5 mL/0.2 g depending on the NADES using MAE.	80% methanol	NADES showed a high yield of curcuminoids and antioxidants compared to 80% methanol.	[33]
*Scutellaria baicalensis* Georgi	Baicalein, scutellarein, wogonin, and oroxylin A (flavonoid aglycones); baicalin, scutellarin, wogonoside, and oroxyloside (flavonoid glycosides)	Proline-citric acid (1:1) with 40% water or citric acid-β-alanine (1:1) with 50% water; for 30 min, extract with UAE at room temperature.	80% methanol and 70% ethanol	The yield of aglycon flavonoids extracted with NADES was 2–6 times that of 80% methanol. However, as compared to aqueous methanol, the number of glycosides extracted with NADES was only 1.5–1.8 times greater.	[34]
*Peumus boldus* Molina	Boldine and phenolics	l-proline-oxalic acid (1:1) with 20% water; 340 rpm, at 50 °C for 50 min.	Methanol and water	The selected NADES was eight times more efficient for extraction of boldine than methanol. No significant differences of TPC in extracts obtained with methanol and NADES.	[32]

Note: MAE = microwave-assisted extraction; HRE = heat reflux extraction; UAE = ultrasonic-assisted extraction; NPCE = negative pressure cavitation extraction.

**Table 5:** Application of DES/NADES to improve bioavailability of plant metabolites.

Bioactive Compounds	Selected DES/NADES	Route	Results	Refs.
Rutin	Proline-glutamic acid	Oral	Improved pharmacokinetics due to increased blood concentration	[62]
Berberine	Proline-malic acid-lactic acid-water (1:0.2:0.3:0.5)	Oral	Improved pharmacokinetics due to increased blood concentration	[61]
Curcumin	Choline chloride-glycerol (1:1)	-	Solubility in gastric and intestinal media was increased	[29]
Caffeine	Choline-glutamine and choline-phenylalanine	Topical	Appropriate for in vitro skin application when formulated as oil-in-water emulsions and gels; increased drug loading while preserving formulation stability	[63]
Calycosin-7-O-β-D-glucoside	Glucose-fructose-sucrose (32:32:5 by weight) and water	Oral	Increased blood concentration	[58]
Hydroxysafflor yellow A (HSYA) and anhydrosafflor yellow B (ASYB)	l-proline-acetamide	Oral	Improved pharmacokinetics due to increased blood concentration	[64]
